# The protective effects of whortleberry extract against cisplatin-induced ototoxicity in rats^☆^

**DOI:** 10.1016/j.bjorl.2017.10.009

**Published:** 2017-11-10

**Authors:** Doğukan Özdemir, Abdulkadir Özgür, Yıldıray Kalkan, Suat Terzi, Levent Tümkaya, Adnan Yılmaz, Metin Çeliker, Engin Dursun

**Affiliations:** a University of Health Sciences, Samsun Health Practices and Research Center, Department of Otorhinolaryngology, Samsun, Turkey; b Recep Tayyip Erdogan University, Medical Faculty, Department of Histology and Embryology, Rize, Turkey; c Recep Tayyip Erdogan University, Medical Faculty, Department of Otorhinolaryngology, Rize, Turkey; d Recep Tayyip Erdogan University, Medical Faculty, Department of Biochemistry, Rize, Turkey

**Keywords:** Ototoxicity, Cisplatin, Whorleberry extract, Ototoxicidade, Cisplatina, Extrato de uva-do-monte

## Abstract

**Introduction:**

Cisplatin is one of the main chemotherapeutic agents used for the treatment of many types of cancer. However, ototoxicity, one of the most serious side effects of cisplatin, restricts its usage.

**Objective:**

We aimed to investigate the protective effects of whortleberry extract against cisplatin-induced ototoxicity by evaluating hearing and histopathological cochlear damage and by measuring the biochemical parameters affected byoxidative stress.

**Methods:**

Forty-eight male rats were included in the study after performing Distortion Product Otoacoustic Emission test to confirm that their hearing levels were normal. The rats were randomly divided into six groups: the control group, the sham group, and, which received only whortleberry extract, only cisplatin, cisplatin + 100 mg whortleberry extract, cisplatin + 200 mg whortleberry extract, respectively. Audiologic investigation was performed by performing the Distortion Product Otoacoustic Emission test at the beginning and at the eighth day of the study. Cardiac blood samples were collected for biochemical analysis, and the rats were sacrificed to obtain cochlear histopathological specimens on the eighth day.

**Results:**

The results revealed that whortleberry protects hearing against cisplatin-induced ototoxicity independent of the dose. However, high doses of whortleberry extract are needed to prevent histopathological degeneration and oxidative stress.

**Conclusion:**

The results obtained in this study show that whortleberry extract has a protective effect against cisplatin-induced ototoxicity.

## Resumo

**Introdução:**

A cisplatina é um dos principais agentes quimioterápicos utilizados para o tratamento de muitos tipos de câncer. No entanto, a ototoxicidade, um dos efeitos colaterais mais graves da cisplatina, restringe seu uso.

**Objetivo:**

Nosso objetivo foi investigar os efeitos protetores do extrato de uva-do-monte contra a ototoxicidade induzida por cisplatina, avaliar o dano auditivo e histopatológico coclear e medir os parâmetros bioquímicos afetados pelo estresse oxidativo.

**Método:**

Foram incluídos no estudo 48 ratos machos após teste de emissão otoacústica evocada por produto de distorção para confirmar que seus níveis de audição eram normais. Os ratos foram divididos aleatoriamente em seis grupos: o grupo controle, o grupo simulado, o que recebeu apenas extrato de uva-do-monte, o que recebeu apenas cisplatina, o que recebeu cisplatina + 100 mg de extrato de uva-do-monte e o que recebeu cisplatina + 200 mg de extrato de uva-do-monte, respectivamente. A investigação audiológica foi feita através do teste de emissão otoacústica de produto de distorção no início e no oitavo dia do estudo. As amostras de sangue cardíaco foram coletadas para análise bioquímica e os ratos foram sacrificados para obtenção de espécimes histopatológicos cocleares no oitavo dia.

**Resultados:**

Os resultados revelaram que o extrato de uva-do-monte protege a audição contra a ototoxicidade induzida por cisplatina, independentemente da dose. No entanto, são necessárias doses elevadas do extrato para evitar a degeneração histopatológica e o estresse oxidativo.

**Conclusão:**

Os resultados obtidos neste estudo mostram que o extrato de uva-do-monte tem um efeito protetor contra a ototoxicidade induzida por cisplatina.

## Introduction

Cisplatin is a chemotherapeutic agent frequently used for the treatment of many types of cancer. Side effects, such as ototoxicity, neurotoxicity, and nephrotoxicity, are cisplatin's dose-limiting side effects. While nephrotoxicity can be prevented by increased hydration and enhanced diuresis, there is still no preventive or curative treatment for ototoxicity. Previous studies showed a worsening of hearing thresholds in 75–100% of patients who received cisplatin. This side effect was more common in the childhood age groups.^1^, ^2^

The cellular and molecular mechanisms of cisplatin-induced ototoxicity have not yet been fully understood. Cisplatin has been presumed to have destructive effects, especially on the outer hair cells, that result from the excessive production of free oxygen radicals in the cochlea.^3^, ^4^ The main cytotoxic effect of cisplatin is believed to occur via the monohydrate cisplatin complex, which reacts with nuclear DNA. Due to the close association of cytotoxicity with oxygen radicals, treatment research has focused on antioxidants for many years.^5^, ^6^

Although bilberry extract has been shown to have a wide spectrum of physiological effects, such as visual improvement, antineoplastic effects, neuroprotective effects, and anti-inflammatory effects, its effects against ototoxicity are unknown in rat experiments. It is believed that these effects appeared because of high levels of anthocyanins in bilberries.^7^, ^8^ Whortleberry (*Vaccinium myrtillus*), which is a species of bilberry, is a potentially promising drug group.^9^

The aim of this study was to investigate the protective effects of whortleberry extract against cisplatin-induced ototoxicity by evaluating hearing and histopathological cochlear damage and by measuring biochemical parameters affected by oxidative stress.

## Methods

The study was approved by the Institutional Ethics Committee for Animal Experiments (n° 2015/19). The rats used in the study were obtained from the experimental animal practice unit of our institution, and the study was carried out at this unit. This work was supported by Recep Tayyip Erdogan University Scientific Research Project Unit (RTEUBAP) under the project number of 2015/9.

### Study protocol

Forty-eight adult male Wistar albino rats ranging in weight from 250 g to 280 g were included in this study after using a Distortion Product Otoacoustic Emission (DPOAE) test to confirm the health of their hearing. All interventional procedures were performed while the rats were under anesthesia, which was induced with intraperitoneally applied 50 mg/kg ketamine hydrochloride (Ketalar^®^, Eczacıbaşı Parke-Davis, İstanbul, Turkey) and 10 mg/kg xylazine hydrochloride (Alfazyne^®^, Alfasan International B.V., Woerden, Holland). The rats’ rectal temperatures were measured regularly while the rats were under anesthesia, and warm blankets that held the rats’ body temperatures at about 35 °C were placed over the rats. The rats with Signal-to-Noise Ratio (SNR) values of 3 dB or higher in three of the five evaluated frequencies in the DPOAE test were considered to have a healthy hearing level. During the study, the rats were kept at the experimental animal unit in 12 h of light and 12 h of darkness each day at a room temperature of 22 ± 3 °C and a humidity of 55–60%. The animals were allowed to consume unlimited food and tap water ad libitum. At every stage of the study, the external auditory canal and the tympanic membranes of the rats were examined otoscopically to exclude other factors that may have influenced the test results, such as cerumen, signs of infection, or tympanic membrane perforation.

The rats were randomly divided into six groups of eight animals. Auditory investigations were performed by obtaining a DPOAE test at the beginning and the eighth day of the study. Study groups were treated as follows: The first group (control group; *n* = 8) was not exposed to any intervention except the DPOAE measurement. The second group (sham group; *n* = 8) received intraperitoneal (i.p.) solvent (distilled water + methanol) for eight days. The third group (whortleberry control group; *n* = 8) received i.p. whortleberry extract (100 mg/kg) for eight days. The fourth group (cisplatin group; *n* = 8) received a single dose of i.p. 16 mg/kg cisplatin (Cisplatin DBL 100 mg/100 mL flacon, Orna İlaç, İstanbul) on the fifth day. The fifth group (cisplatin + 100 mg/kg whortleberry group; *n* = 8) received i.p. whortleberry extract (100 mg/kg) for eight days and a single dose of i.p. 16 mg/kg cisplatin on the fifth day. The sixth group (cisplatin + 200 mg/kg whortleberry group/*n* = 8) received i.p. whortleberry extract (200 mg/kg) for eight days and a single dose of i.p. 16 mg/kg cisplatin on the fifth day.

### Preparation of the whortleberry extract

Whortleberry extract (Bilberry [*V. myrtillus*] Herbal Liquids, Health Aid, England) containing a mix of half distilled water and half ethanol with 330 mg/mL of whortleberry extract was used as a nutritional support product. The product was extra-diluted in sterile conditions with a mix of half distilled water and half ethanol to achieve a whortleberry concentration of 50 mg/mL.

### Measurement of DPOAE

DPOAE recording was performed using an Echoport ILO292-II (Otodynamics, Hatfield, England) set to DPOAE mode in a quiet room. After keeping the head of each rat in the horizontal position, an infant-size probe was attached to the external auditory canal of the animals. Measurements were obtained after confirming that the device was in the proper measurement position with the appropriate configuration of the probe indicator and stimulus waveform. The ratio between frequencies f2 and f1 (f2/f1) was held at 1.22. The intensity of the stimulus was taken as L1 for frequency f1 and as L2 for frequency f2. The difference between the levels of L1 and L2 was kept at 10 Db SPL (sound pressure level) (L1 = 65 dB SPL, L2 = 55 dB SPL). The results were shown in the geometric mean of the primary tones (f1 and f2). Otoacoustic emissions were created using two different speakers for the two stimuli (f1 and f2) in the external ear canal. DPOAEs were measured at the 2f1-f2 frequency with a microphone on the outer ear canal. The resulting otoacoustic emissions were recorded with the geometric mean of f1 and f2 at 2000, 3000, 4000, 6000, and 8000 Hz. The test duration was about 60 s. The values of DPOAE amplitudes that were 3 dB above the noise threshold were considered significant. SNR frequency curves were drawn. The SNR values of all groups obtained during the DPOAE test were compared within and between the groups.

### Biochemical evaluation

Cardiac blood samples of all groups were taken while the animals were under anesthesia at the end of the study. After centrifuging these samples at 3500 rpm for 5 min, the Total Antioxidant Status (TAS) and Total Oxidative Stress (TOS) levels of the samples were measured with an autoanalyzer (Abbott C16000, Abbott Diagnostics, Abbott Park, IL, USA) using Rel Assay TAS and Total Oxidant Stress Test Kits (Mega Tıp, Gaziantep, Turkey) at the biochemistry laboratory of our institution.

The TAS measurement method was based on the principle that the color of colored cationic radical 2.2′-azino-bis (3-ethylbenzothiazoline-6-sulphonic acid) (ABTS) is lost as a result of ABTS's reduction in proportion to the total concentration of antioxidant molecules. The results of the measurements were expressed as mmol Trolox equivalent/L.^10^

In the measurement of total oxidant level, the colorimetric method was used, which was based on the principle of the cumulative oxidation of the ferrous ion to the ferric ion by the oxidant molecules.^11^

Oxidative stress index (OSI) was calculated using the formula:

$$\text{OSI}=\frac{\text{TOS}\;(\mu {\text{mol H}}_{2}{\text{O}}_{2}\;\text{equivalent}/\text{L})}{\text{TAS}\;(\mu \text{mol}\;\text{Trolox}\;\text{equivalent}/\text{L})}$$

### Histopathological evaluation

All animals were sacrificed for histopathological evaluation after the last DPOAE test and blood sample collection. The temporal bone was dissected, and the bulla was opened. The lateral wall of the cochlea was removed, and the cochlea was fixed with a 2.5% glutaraldehyde solution after a slow injection. The temporal bones were kept in the same solution at +4 °C overnight. After fixation, the temporal bones were waited in a 10% EDTA solution at +4 °C for ten days for decalcification. After this procedure, the cochlear specimens were dehydrated with ethanol and embedded in paraffin blocks. Then, sections with a thickness of 5 μm were prepared and stained with hematoxylin-eosin. At least fifteen sections from each rat cochlea were examined. Signs of degeneration, such as dilation, apoptotic cells, cell degeneration, nerve degeneration, and cytoplasmic vacuolization, were scored separately by a histopathologist who was blinded to group information. Each rat was graded on a five-point scale that ranged from 0 to 4 for signs of degeneration (0: normal, 1: mild, 2: moderate, 3: moderate to severe, and 4: severe).

### Statistical analysis

The data was analyzed with SPSS version 15.0 for Windows (SPSS Inc., Chicago, IL, USA). The results were presented as means ± standard error of measurement. All data were analyzed with a one-way analysis of variance post hoc Bonferroni test. A *p*-value less than 0.05 was considered to indicate a significant difference.

## Results

### DPOAE results

A DPOAE test was performed on the animals in all groups before the onset of the study, and the base SNR values were obtained at each frequency. On the eighth day of the study, a DPOAE test was performed again, and the SNR values were recorded. The SNR values, which had been obtained for 2000, 3000, 4000, 6000, and 8000 Hz at beginning and end of the study, were compared within and between groups. There was no significant difference between groups in the DPOAE measurements performed on the first day (*p* > 0.05). However, compared with the first test, the SNR values of the cisplatin group were significantly lower at all frequencies on the eighth day (*p* < 0.01). Similarly, the SNR values of the cisplatin group were found to be lower than those of the other groups at all frequencies on the eighth day (*p* < 0.015 for 2000, 3000, and 4000 Hz, *p* < 0.001 for 6000 and 8000 Hz).

There was no significant decrease in SNR values on the eighth day in the groups that received cisplatin with whortleberry extract (*p* > 0.05). These results show that whortleberry extract has a protective effect against cisplatin-induced ototoxicity. However, there was no significant difference between the groups treated with cisplatin + 100 mg/kg whortleberry extract and cisplatin + 200 mg/kg whortleberry extract in terms of hearing protection (*p* > 0.05).

**Figure 1:**
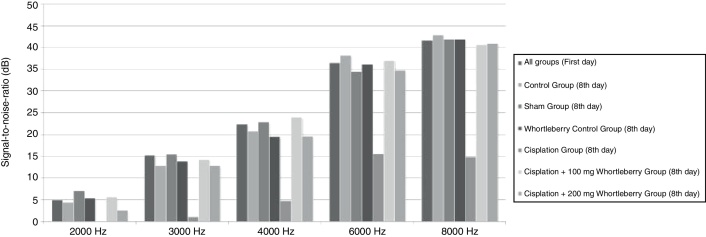
Average signal-to-noise ratio values for all groups.

The average SNR values of all groups for the first day and the average SNR values according to each group for the eighth day are presented in Fig. 1.

### Biochemical evaluation results

The TAS values obtained from the biochemical evaluation are summarized in Table 1. According to the obtained data, the TAS values of the whortleberry control group (*p* < 0.001) and the cisplatin + 200 mg whortleberry group (*p* = 0.002) were significantly higher than those of the control group. There was no significant difference between the other groups and the control group.

**Table 1:** Total antioxidant status values according to groups.

Groups	Total antioxidant status Mean ± standard deviation (min–max)
Control group	1.12 mmoL Trolox equivalent/L ± 0.10 (0.96–1.60)
Sham group	1.11 mmoL Trolox equivalent/L ± 0.11 (0.98–1.27)
Whortleberry control group	1.45 mmoL Trolox equivalent/L ± 0.16 (1.12–1.60)
Cisplatin group	1.04 mmoL Trolox equivalent/L ± 0.05 (0.97–1.11)
Cisplatin + 100 mg/kg whortleberry group	1.21 mmoL Trolox equivalent/L ± 0.12 (1.01–1.36)
Cisplatin + 200 mg/kg whortleberry group	1.34 mmoL Trolox equivalent/L ± 0.03 (1.27–1.38)

The TOS data of the groups are summarized in Table 2. The TOS values of the cisplatin group (*p* = 0.001) and the cisplatin + 100 mg whortleberry group (*p* = 0.015) were significantly higher than those of the control group (*p* < 0.05). There was no significant difference between the other groups and the control group (*p* > 0.05).

**Table 2:** Total oxidative stress values according to groups.

Groups	Total oxidative stress Mean ± standard deviation (min–max)
Control group	7.40 μmoL H_2_O_2_ equivalent/L ± 1.56 (5.60–9.56)
Sham group	12.88 μmoL H_2_O_2_ equivalent/L ± 6.57 (6.48–23.76)
Whortleberry control group	10.71 μmoL H_2_O_2_ equivalent/L ± 4.21 (5.79–18.73)
Cisplatin group	22.49 μmoL H_2_O_2_ equivalent/L ± 10.73 (11.54–40.09)
Cisplatin + 100 mg/kg whortleberry group	18.90 μmoL H_2_O_2_ equivalent/L ± 5.38 (8.95–24.37)
Cisplatin + 200 mg/kg whortleberry group	14.67 μmoL H_2_O_2_ equivalent/L ± 7.56 (8.85–31.41)

The calculated OSI values of the groups are summarized in Table 3. The OSI value of the cisplatin group (*p* < 0.001) and the cisplatin + 100 mg whortleberry group (*p* = 0.031) were significantly higher than those of the control group. There was no significant difference between the other groups and the control group (*p* > 0.05).

**Table 3:** Oxidative stress index values according to groups.

Groups	Oxidative stress index Mean ± standard deviation (min–max)
Control group	6.72 ± 1.84 (4.55–9.96)
Sham group	11.66 ± 5.80 (5.50–20.96)
Whortleberry control group	7.41 ± 2.71 (3.71–11.93)
Cisplatin group	21.54 ± 9.94 (11.54–39.31)
Cisplatin + 100 mg/kg whortleberry group	16.02 ± 5.42 (6.73–23.14)
Cisplatin + 200 mg/kg whortleberry group	11.03 ± 5.76 (6.65–23.62)

### Results of the histopathological evaluation

**Figure 2:**
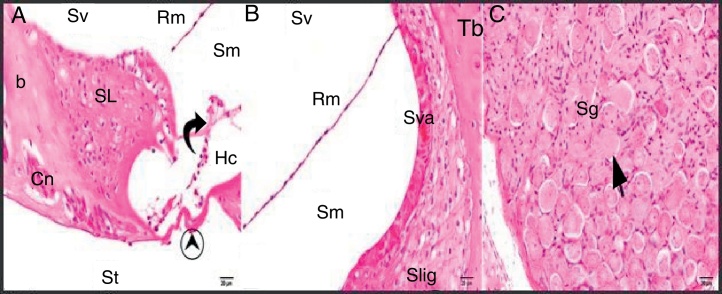
Control group: SL, spiral limbus; b, bone; Rm, Reissner's membrane; Sv, scala vestibule; Sm, scala media; St, scala tympani; round arrowhead, basilar membrane; curved arrow, tectorial membrane; Hc, inner hair cell; Cn, cochlear nerve; Sva, stria vascularis; Slig, spiral ligament; Sg, spiral ganglion; black arrow, spiral ganglion cells (hematoxylin eosin stain, ×40).

The general structure of the control group showed normal histological findings in a light microscopic evaluation (Fig. 2A–C).

The sham and whortleberry control groups similarly showed normal histological structures in the light microscopic examination. No positive or negative effects on cellular structures and morphology were identified in either group.

**Figure 3:**
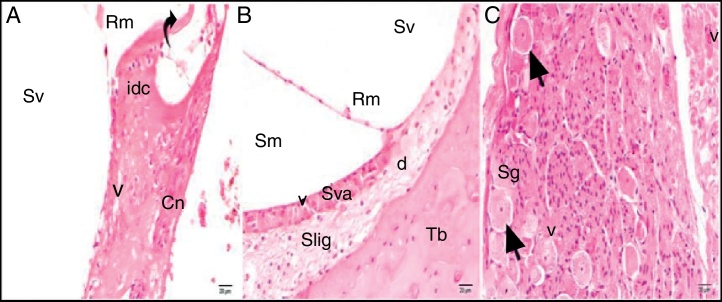
Cisplatin group: Rm, Reissner's membrane; Sv, scala vestibule; Sm, scala media; St, scala tympani; round arrowhead, basilar membrane; d, degeneration and dilatation; v, vacuolization; curved arrow, tectorial membrane; Cn, cochlear nerve; Sva, stria vascularis; Slig, spiral ligament; Sg, spiral ganglion; black arrow, spiral ganglion cells; (hematoxylin eosin stain, ×40).

The light microscopic evaluation of the cisplatin group revealed that the general structure of the tissue had deteriorated. Increased cytoplasmic vacuolization and degenerative cell separation was observed. Although the organ of Corti had maintained its integrity, the extension and cell body of the inner and outer hair cells showed local swelling and degenerative separation. The cells’ nuclei were observed to be slightly acidophilic, and their cytoplasm showed vacuolization. The tectorial membrane was thickened with normal morphology, whereas Reissner's membrane showed swollen cells and cellular separation. Although the nuclei of the supporting cells were slightly basophilic, a small amount of vacuolization was observed in their cytoplasm. Vacuolization in the spiral limbus region showed separations as dilatation (Fig. 3A). Cells forming the stria vascularis and spiral ligament were pathologic compared with the control group in terms of the array and nucleus view. Dilatations and loss of nucleus due to cell degeneration were detected. Increased vacuolization and some degree of degenerated cells were seen at the stria vascularis and spiral ligament. There were dilated cells in the spiral limb, and they were intensely acidophilic (Fig. 3B). The spiral ganglion and cochlear nerve fibers were somewhat degenerated. Increased vacuolization and axonal degeneration were identified. Deformation of cell bodies was seen in pseudounipolar neurons with a small amount of vacuolization (Fig. 3C).

**Figure 4:**
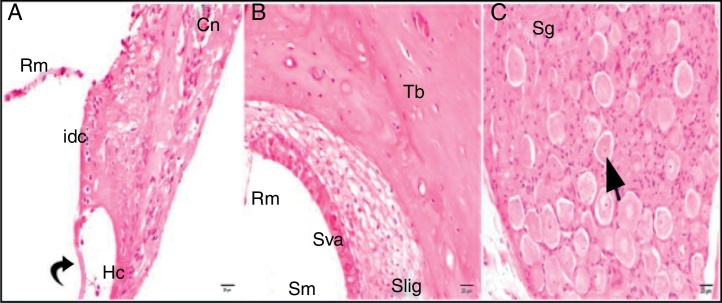
Cisplatin + 100 mg/kg whortleberry group; Rm, Reissner's membrane; Sv, scala vestibule; Sm, scala media; St, scala tympani; round arrowhead, basilar membrane; idc, interdental cell; Hc, inner hair cell; d, degeneration and dilatation; v, vacuolization; curved arrow, tectorial membrane; Cn, cochlear nerve; Sva, stria vascularis; Slig, spiral ligament; Sg, spiral ganglion; black arrow, spiral ganglion cells; (hematoxylin eosin stain, ×40).

**Figure 5:**
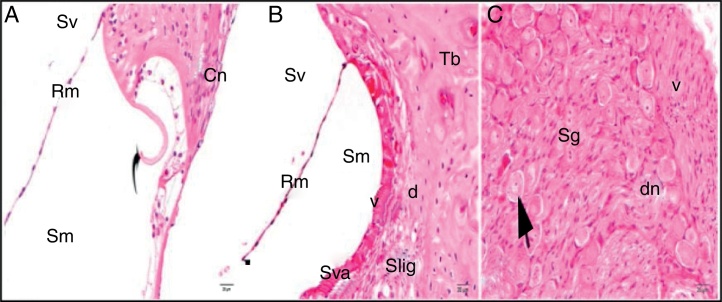
Cisplatin + 200 mg/kg whortleberry group; Rm, Reissner's membrane; Sv, scala vestibule; Sm, scala media; St, Scala tympani; round arrowhead, basilar membrane; idc, interdental cell; Hc, inner hair cell; d, degeneration and dilatation; v, vacuolization; curved arrow, tectorial membrane; Cn, cochlear nerve; Sva, stria vascularis; Slig, spiral ligament; Sg, spiral ganglion; black arrow, spiral ganglion cells; (hematoxylin eosin stain, ×40).

Although the comparisons of the cisplatin + 100 mg/kg whortleberry and cisplatin + 200 mg/kg whortleberry groups with the control group showed degeneration findings, these findings were not severe as those in the cisplatin group (Figure 4, Figure 5).

The results of the histopathological blind grading of the groups according to the dilatation, apoptotic cell, nerve degeneration, cellular degeneration, and cytoplasmic vacuolization findings are summarized in Table 4. According to the histopathological blind grading results, no significant difference was observed between the control group and the sham and the whortleberry control groups in terms of degeneration findings (*p* > 0.05). When the control group was compared with the cisplatin and the cisplatin + 100 mg/kg whortleberry groups, there were significant differences in terms of all evaluation criteria (*p* < 0.001).

**Table 4:** Histopathological blind grading findings of each group.

Groups	Histopathological Blind Grading Mean ± standard deviation (min–max)				
	Cytoplasmic vacuolization	Cellular degeneration	Dilatation	Apoptotic cell	Nerve degeneration
Control group	0.25 ± 0.46 (0–1)	0.13 ± 0.36 (0–1)	0.13 ± 0.36 (0–1)	0.63 ± 0.52 (0–1)	0.25 ± 0.46 (0–1)
Sham group	0.38 ± 0.52 (0–1)	0	0.25 ± 0.46 (0–1)	0.50 ± 0.53 (0–1)	0.25 ± 0.46 (0–1)
Whortleberry control group	0.75 ± 0.71 (0–2)	0.88 ± 0.99 (0–2)	0.75 ± 0.89 (0–2)	0.50 ± 0.53 (0–1)	0.38 ± 0.52 (0–1)
Cisplatin group	3.63 ± 0.52 (3–4)	3.13 ± 0.35 (3–4)	2.63 ± 0.74 (2–4)	3.00 ± 0.00 (3–3)	2.50 ± 0.53 (2–3)
Cisplatin + 100 mg/kg whortleberry group	1.75 ± 0.46 (1–2)	1.63 ± 0.52 (1–2)	1.75 ± 0.46 (1–2)	2.75 ± 0.46 (2–3)	2.13 ± 0.83 (1–3)
Cisplatin + 200 mg/kg whortleberry group	0.88 ± 0.64 (0–2)	0.88 ± 0.83 (0–2)	0.50 ± 0.53 (0–1)	1.00 ± 0.76 (0–2)	1.50 ± 0.76 (0–2)

## Discussion

Cisplatin is a highly effective antineoplastic agent that is widely used in the treatment of head and neck tumors. However, serious side effects, such as ototoxicity, myelotoxicity, nephrotoxicity, peripheral neuropathy, and gastrointestinal toxicity, limit the clinical use of cisplatin. There have been promising studies aiming to reduce the ototoxic effects of cisplatin, but there is still no treatment that completely prevents cisplatin's ototoxicity.^12^, ^13^ The results obtained in this study showed that whortleberry extract, which has been known as a potent antioxidant, showed a protective effect against cisplatin-induced ototoxicity in the audiological and histopathological findings of rats. This study also showed biochemical evidence that whortleberry extract has a protective effect against oxidative stress, which has been considered one of the mechanisms of the formation of cisplatin ototoxicity.

Various doses and applications to produce cisplatin ototoxicity have been examined in previous documented studies.^14^, ^15^ To induce ototoxicity in the rats in our study, we preferred to use a single dose of 16 mg/kg cisplatin, which, as had been detected in our earlier study, was the optimal dose that could create ototoxicity without disturbing the overall state of the rat. The decreased emission response found using a DPOAE test on the eighth day of the study showed that our cisplatin administration was sufficient for experimental ototoxicity formation. In addition, there were no abnormalities caused by the administration of cisplatin or the other substances that required the exclusion of the rats from the study.

Several antioxidant substances, such as lycopene, curcumin, and ginkgo biloba, have been studied for their potential to prevent cisplatin ototoxicity, and these substances have been shown to have protective effects against ototoxicity.^14^, ^16^, ^17^ Bilberry species, which are known as potent antioxidants, have been shown to exhibit physiological effects, such as anticancer effects, neuroprotective effects, anti-inflammatory effects, and enhancement of vision, in rats. Additionally, these species can reduce oxidative stress.^9^, ^18^ A previous study showed that orally administered bilberry extract had a protective effect against ototoxicity.^19^ In our study, extract of whortleberry, which is a species of bilberry, was diluted and applied intraperitoneally. In addition, the effects of different doses were compared. Studies about the i.p. application of bilberry are very limited. We did not have any complications during i.p. whortleberry extract application.

According to the DPOAE responses in our study, whortleberry extract had protective effect against cisplatin-induced ototoxicity in both low and high dose applications. The ototoxic effects of cisplatin were identified on the cellular level during the histopathological evaluation, and the low dose of whortleberry extract was not able to prevent this histopathological degeneration. However, high doses of the extract prevented the degeneration. The preservation of hearing in the low-dose whortleberry extract group suggests that even if the whortleberry extract does not provide complete protection against histopathologic degeneration, whortleberry can prevent enough degeneration to prevent hearing impairment.

Studies about the effects of bilberry extract on oxidative stress have shown that bilberry reduces oxidative stress.^20^, ^21^ Oxidative stress parameters, which have been considered one of the causes of cisplatin ototoxicity, were therefore also assessed biochemically in our study. According to the data we obtained, cisplatin increases TOS and OSI. Although low doses of whortleberry extract cannot reduce the oxidative stress that cisplatin triggers, whortleberry exhibits an antioxidant effect in high doses and reduces the OSI accordingly.

## Conclusion

The data we obtained revealed that whortleberry protects hearing against cisplatin-induced ototoxicity and avoids cellular degeneration in high doses. In addition, in high doses, whortleberry can significantly reduce oxidative stress, which is thought to be one of the mechanisms of ototoxicity. In light of the data we obtained, there is a need for new extensive clinical studies to determine the effectiveness of whortleberry in greater detail.

## Funding

This work was supported by Recep Tayyip Erdogan University Scientific Research Project Unit (RTEUBAP) under the project number of 2015/9. Compliance with ethical standards; the study was performed in accordance with the 2011 Guide for the Care and Use of Laboratory Animals.

## Conflicts of interest

The authors declare no conflicts of interest.
